# Relationship Between Depression and Decreased Activity Level and Cognitive Impairment in Patients with Diabetes Mellitus Type 2 and/or Atrial Fibrillation

**DOI:** 10.3390/jcm14020563

**Published:** 2025-01-16

**Authors:** Marius Militaru, Daniel Florin Lighezan, Cristina Tudoran, Flavia Zara, Adina Bucur, Anda Gabriela Militaru

**Affiliations:** 1 Department of Neuroscience, Discipline of Neurology II, University of Medicine and Pharmacy “Victor Babes”, E. Murgu Square, Nr. 2, 300041 Timisoara, Romania; marius.militaru@umft.ro; 2 Emergency City Hospital Timisoara, Gheorghe Dima Street, Nr. 5, 300254 Timisoara, Romania; dlighezan@umft.ro (D.F.L.); militaru.anda@umft.ro (A.G.M.); 3 Centre of Advanced Research in Cardiology and Hemostasology, University of Medicine and Pharmacy “Victor Babes” Timisoara, E. Murgu Square, Nr. 2, 300041 Timisoara, Romania; 4 Department of Internal Medicine I, Discipline of Medical Semiology I, University of Medicine and Pharmacy “Victor Babes”, E. Murgu Square, Nr. 2, 300041 Timisoara, Romania; 5 Department VII, Internal Medicine II, Discipline of Cardiology, University of Medicine and Pharmacy “Victor Babes” Timisoara, E. Murgu Square, Nr. 2, 300041 Timisoara, Romania; 6 Centre of Molecular Research in Nephrology and Vascular Disease, University of Medicine and Pharmacy “Victor Babes” Timisoara, E. Murgu Square, Nr. 2, 300041 Timisoara, Romania; 7 County Emergency Hospital “Pius Brinzeu”, L. Rebreanu, Nr. 156, 300723 Timisoara, Romania; 8 Department of Microscopic Morphology, University of Medicine and Pharmacy “Victor Babes”, E. Murgu Square, Nr. 2, 300041 Timisoara, Romania; flavia.zara@umft.ro; 9 Department of Pathology, Emergency City Hospital, Gheorghe Dima Street, Nr. 5, 300254 Timisoara, Romania; 10 Department of Functional Sciences, Discipline of Public Health, Centre for Translational Research and Systems Medicine, University of Medicine and Pharmacy “Victor Babes” Timisoara, E. Murgu Square, Nr. 2, 300041 Timisoara, Romania; bucur.adina@umft.ro

**Keywords:** cognitive decline, dementia, type 2 diabetes mellitus, atrial fibrillation, SCORE2, memory tests

## Abstract

**Background:** The interdependence between type 2 diabetes mellitus (DM-2), atrial fibrillation (AF), and cognitive decline (CD)/dementia is a debated topic. In this study, we highlighted the influence of DM-2 and FA individually and in association on the severity of CD/dementia. **Methods:** This study comprises 248 patients with very high cardiovascular risk (VHCVR) according to Systematic Coronary Risk Evaluation (SCORE2), of whom 184 had DM-2 and/or AF, and 64 were age-matched controls (without DM-2/AF), admitted to the Municipal Hospital Timisoara. **Results:** Mini-Mental-State-Examination (MMSE), Montreal Cognitive Assessment (MoCA), Activities of Daily Living Score (ADL), and Instrumental Activities of Daily Living Score (IADL) were significantly decreased, and Geriatric Depression Scale (GDS-15) increased in patients with DM-2 and AF in comparison to controls (*p* < 0.05), with the subjects with DM-2 and AF having more severe CD compared to those with only one of these two pathologies. The logistic regression model showed that the risk of CD (MMSE < 27) or dementia (MMSE < 24) increased significantly in patients with DM-2 and/or AF depending on the SCORE2 values, ADL, and GDS-15. In DM-2 and/or AF patients, an increase of 1% in SCORE2 was associated with an elevation of 2.40% in the odds of CD and of 4.30% of dementia. In these patients, depression (GDS score) increased the risk of CD by 36.3%, and if ADL improved, the risk of CD decreased by 44.0%. **Conclusions:** Our findings suggest a direct association between CD, DM-2, and AF with SCORE2, cognitive parameters, ADL, and depression. In patients with DM-2 and/or AF, it is important to identify subclinical CD to prevent the evolution to dementia.

## 1. Introduction

Considering that the general population’s life expectancy is extending worldwide [1,2], the number of elderly people with multiple age-related degenerative disorders, including cardiovascular diseases (CVD), cognitive decline (CD), and dementia, is progressively increasing, the healthcare systems will have to make significant efforts to cover the elevated costs [2,3]. In some situations, CD may remain undiagnosed, especially in elderly patients with associated comorbidities [4].

Atrial fibrillation (AF) is worldwide the most common cardiac arrhythmia, with important repercussions on health services and a significant impact on both primary and secondary care. Several studies have highlighted that AF increases the hospitalization rate for CVD decompensation, the risk of developing chronic heart failure (CHF), and the mortality in the affected patients. The number of patients with AF continues to increase parallel with the general population′s aging, the augmentation of CVD prevalence with numerous associated comorbidities, and the increased availability of new diagnostic technologies [4,5]. It is assumed that the incidence of AF will increase by almost 150% in the next four decades in the general population [6,7]. There is more and more evidence demonstrating that AF is an important risk factor for the development of cognitive decline (CD) and dementia. Numerous recent studies and meta-analyses highlighted the association between AF and CD and dementia, even in the absence of a previous stroke [6,7,8,9]. It has been proven that the incidence of dementia will double with each increase in age by 5.9 years, reaching >75 million people worldwide by 2030 and >135 million by 2050 [6,7]. The elevated number of patients with AF and dementia in the coming years will bring a significant increase in healthcare efforts and costs [6,7].

The link between AF and cognitive dysfunction is complex and multifactorial; therefore, several mechanisms have been proposed, with both diseases having in common the association of multiple factors, such as advanced age, the presence of diabetes mellitus (DM), systemic hypertension (SH), obesity, sleep apnea, hyperlipidemia, CHF, the development of chronic kidney disease (CKD), chronic coronary syndrome (CCS), excessive alcohol consumption, and lack of physical activity [6,10,11]. Among these mechanisms, besides the ischemic stroke, inflammation, cerebral hypoperfusion due to AF, systemic atherosclerosis, cerebral atrophy, microhemorrhages, and associated vascular diseases are involved. All these factors can accelerate the evolution towards CD [6,12].

DM type 2 (DM-2) also represents an important global health problem because the number of affected patients is constantly increasing. In 2019, 463 million people (9.3%) were diagnosed with DM-2 worldwide, but it is estimated that this number will increase to 578 million (10.2%) by 2030 and to 700 million people (10.9%) by 2045 [13,14]. DM-2 is a metabolic imbalance, triggering many complications such as diabetic nephropathy, peripheral neuropathy, and diabetic retinopathy and also exerting an important role in the development of CVD and peripheral arterial disease (PAD), resulting in a low quality of life for these patients [13,15].

Numerous studies have documented a direct link between DM-2, cognitive impairment, and dementia, with multiple changes in frontal executive and visuospatial function, verbal fluency, complex motor skills, attention, and processing speed [13,16]. For good functioning, the neurons depend on the glucose metabolism in the brain. Some brain changes, such as reduced grey matter density, hippocampal lesions, and atrophy, associated with changes in glucose metabolism could contribute to neurocognitive dysfunction in these patients [13,17]. In DM-2 patients, several mechanisms, such as insulin resistance, hyperglycemia, endothelial dysfunction, increased oxidative stress, insulin deficiencies, and inflammation, could lead to CD and dementia [13,18]. Changes in insulin may interfere with the degradation of amyloid beta (Aβ), a key pathological feature of Alzheimer’s disease (AD).

Various cardiovascular risk factors (CVRFs), such as SH, DM-2, obesity, smoking, and hyperlipidemia, may significantly contribute to the development of CD and dementia [1].

Considering the increasing prevalence of DM-2 and AF, together with the aging of the global population, it is assumed that CD related to these pathologies would significantly impact the health-care system worldwide. Therefore, a precocious and comprehensive evaluation of patients with CVD, especially with AF, and DM-2 concerning the timely diagnosis of CD and dementia should be performed to increase the level of awareness of the medical staff regarding the development of specific therapeutic and psychological management strategies [2]. Nowadays, various methods are available for an early diagnosis of CD, and for these patients′ follow-up, since CD and, subsequently, dementia exert an important global impact both from clinical and socio-economical points of view [1].

The main purpose of this research was to highlight the impact of DM-2 and AF on the severity of CD/dementia in individuals with multiple CVRFs. A second aim was to establish the importance of an early diagnosis of CD in patients with DM-2 and/or AF for initiating an appropriate treatment. A third aim was to determine the impact of depression and quality of life on the evolution of CD in this category of patients. We consider that it is necessary to evaluate the cognitive function in patients with DM-2 and AF as well as to recognize the small changes related to lifestyle, along with a proper treatment of these patients to prevent or delay CD.

## 2. Materials and Methods

### 2.1. Study Population

From the patients admitted between 1 June 2021 and 30 January 2024 in the internal medicine department of the Municipal Emergency Hospital Timisoara Romania for the evaluation of their previously diagnosed CVD or neurologic pathologies, we included in our study group 248 patients, which, according to Systematic Coronary Risk Evaluation (SCORE2), had very high cardiovascular risk (VHCVR): 184 patients also had DM-2 and/or AF, and 64 controls were without DM-2 and AF. All participants showed progressive memory loss suggesting CD but had not been previously diagnosed with CD or dementia, nor were they treated for these pathologies. All patients had chronic, stable CVD and were under optimal medical therapy. DM-2 was treated with oral glucose-lowering medication, and AF patients were under chronic anticoagulation therapy.

According to the presence of AF and/or DM-2, we divided our patients into four groups: group A—63 patients with DM-2, group B—60 patients with DM-2 and AF, group C—61 patients with AF but without DM-2, and group D—64 age-matched controls with VHCVR without AF and DM-2.

Inclusion criteria included the following: (1) age over 45 years, considering that CD is seldomly encountered before this age; (2) history of CVD and/or AF or DM-2; and (3) signs of cognitive impairment without a previous diagnosis of CD or dementia. Exclusion criteria included the following: (1) uncontrolled DM-2 or necessitating insulin therapy; (2) acute decompensate CVD: acute heart failure, acute myocardial infarction, etc.; (3) uncontrolled SH; (4) other acute or decompensated pathologies; (5) unable or unwilling to sign the informed consent form; and (6) with previously diagnosed cognitive impairment or dementia and under treatment for these conditions.

All patients underwent a detailed clinical examination, followed by cardiologic and neurologic evaluation. The clinical assessment performed for all patients included in the study consisted of medical history, clinical examination, registration of heart rate (HR), systolic blood pressure (SBP), and diastolic blood pressure (DBP). Laboratory data were obtained for all patients, including a lipid profile, N-terminal pro–B-type natriuretic peptide (NT-pro-BNP) levels, and other associated CVRFs. AF was evaluated according to guidelines recommendations [5,19]. DM-2 was determined according to medical history, basal blood glucose (BBG) levels, and glycated hemoglobin. We performed in all patients rest electrocardiogram (ECG), ankle-brachial index (ABI), and carotid artery Doppler ultrasonography to determine intima–media thickness (IMT). Cerebral computed tomography was not mandatory for the diagnosis of cognitive decline. Transthoracic echocardiography (TTE) was performed to determine the left ventricular (LV) and left atrial (LA) dimensions and function. The presence and severity of CD were assessed with the following scales: Mini-Mental State Examination (MMSE) [20,21], Montreal Cognitive Assessment (MoCA) [22,23], Activities of Daily Living (ADL) [24], Instrumental Activities of Daily Living (IADL) [25], and Geriatric Depression Scale (GDS-15) [26,27,28]. In patients with AF and those with DM-2 and AF, we calculated the CHA_2_DS_2_-VASc score. All patients in our study had a SCORE2 assessment, a risk prediction algorithm to estimate the 10-year fatal and non-fatal CVD risk.

The study was conducted according to the guidelines of the Declaration of Helsinki and approved by the Institutional Review Board of Municipal Emergency Hospital Timisoara, Romania nr. E1518/17.03.2021.

### 2.2. Clinical Assessments

#### 2.2.1. Transthoracic Echocardiography (TTE)

To perform the TTE examination, a General Electric Vivid F9 Expert 4D BT 12, VE93611 CISPR 11, Group 1, Class A ultrasound system (GE Vingmed Ultrasound AS, Strandpromenaden 45, 3191 Harten, Norway) device with an M5S MHz transducer was employed according to the guidelines recommendations [29]. All TTE exams were performed on the same ultrasound system and by the same skilled examiner. The assessments were performed in the two-dimensional 2D method to evaluate the dimensions and functions of LA and LV. From a 4-chamber view, in end-systole, we measured the diameter of the LA. We measured the standard diameters and volumes of cardiac structures. Using the modified Simpson’s rule, LV performance was assessed by measuring LV ejection fraction (LVEF). With the pulsed Doppler method from an apical window, we determined the trans-mitral flow velocities to quantify the maximum velocity of the early filling wave (E-wave) by placing the probe in a parallel alignment with the mitral blood flow [29].

#### 2.2.2. Intima–Media Thickness (IMT)

The assessment and measurement of the IMT of the common carotid artery (CCA) was carried out using a General Electric Vivid E9 ultrasound system with a 9L MHz transducer by the same examiner. IMT was measured at the level of the distal wall of CCA, at 1 cm from the carotid bulb. Ten measurements were recorded for each patient, and average values were calculated [2].

#### 2.2.3. Ankle Brachial Index (ABI)

The ABI test is a non-invasive and quick way to check the presence of peripheral arterial disease (PAD). The ABI represents a ratio between the blood pressure measured at the ankle and the level of the upper arm. An ABI score of less than 0.90 may indicate a narrowing of the arteries that reduces blood flow to the legs, is considered abnormal, and indicates PAD. An ABI score between 0.91 and 0.99 may point to borderline PAD, and an ABI score between 1.0 and 1.4 may suggest that the patient does not have PAD [30].

#### 2.2.4. Triglyceride-Glucose Index (TyG)

TyG is a marker correlated with lipotoxicity and glucotoxicity [2,30]. A close connection has been demonstrated between the TyG and DM-2, stroke, CVD, SH, and endothelial dysfunction [2,5]. The (TyG) is calculated with the following formula: ln [BBG (mg/dL) × TG (mg/dL)/2], where TG means fasting triglyceride levels. The normal cut-off values of TyG vary widely between 4 and 8 [2,5,31].

#### 2.2.5. Montreal Cognitive Assessment (MoCA)

CD can be broadly assessed using the MoCA scale. This test is performed in approximately 10 min, and the maximum score of this scale is 30 points. This scale assesses several cognitive functions: orientation, language, attention, visual-spatial ability, working memory, concentration, executive functions, short-term memory recall task, copying a three-dimensional cube, and a two-item verbal abstraction task with a phonemic fluency task. A score below 27 indicates a minor cognitive impairment, and values below 24 signify moderate to severe cognitive impairment and dementia [2,4,22,23].

#### 2.2.6. Mini-Mental State Examination (MMSE)

The MMSE scale is widely employed for cognitive disorders screening, for the follow-up of a patient’s cognitive level over time, and to assess the progression and severity of cognitive impairment. The MMSE scale quantifies cognitive functions such as orientation, attention, recall, calculation, language manipulation, and construction practices [2,4,20,21]. The maximum score is 30 points, but this score has to be analyzed according to age and level of education [2,4,20,21,32]. A score between 24–27 means mild to moderate CD, and under 24 indicates dementia [2,4,20,21,32,33,34].

#### 2.2.7. Activities of Daily Living (ADL)

The ADL score is widely used in medical care to assess the patient’s daily care activities. Its administration takes 10 min, and the maximum score is 10 points. Five basic areas are evaluated: eating, transferring and mobility, dressing, maintaining continence, and personal hygiene [2,4,35,36]. The ADL scale evaluates the ability or incapacity, especially of elderly patients with disabilities, to take care of themselves and determines their daily functional status [2,4,35]. A score of 10 points means high functionality, and a score of 0 points means low functionality [2,4,36].

#### 2.2.8. Instrumental Activities of Daily Living (IADL)

The IADL scale is widely used to assess the capacity of daily care and functioning. It consists of questions addressed to the patient or is in a written form that should be completed by the patient. It takes about 10 min to be completed, and the maximum score is 8 points. To assess the more complex activities required for normal functioning, the IADL scale investigates eight domains: cooking, financial management, and shopping. A maximum score of 8 represents high functionality, and a minimum score of 0 represents low functionality [2,4,24,25].

#### 2.2.9. Geriatric Depression Scale (GDS-15)

The GDS scale is a scale used to assess the presence of elements of depression in the population. The GDS scale can be used in younger, middle-aged, and elderly patients. The short scale with 15 questions (GDS-15) is employed more frequently, allowing an easier evaluation, and in our study, we used this version. The GDS-15 scale is easier to apply in patients without CD but also in those with cognitive impairment, for whom a multi-question assessment would be difficult [2,4,31,32,33,34,35]. This test takes approximately 10–15 min, and the patients are asked to answer to 15 questions. The GDS-15 is a short questionnaire with a sensitivity of 92% and a specificity of 89%, and it is interpreted according to age and educational level [2,4,31,32,33,34,35]. The obtained scores indicate the presence or absence of depression and/or cognitive impairment. A score between 12–15 points suggests severe depression, one between 9–11 points defines moderate depression, a value between 5–8 points means mild depression, and between 0–4 points is considered normal [2,4,26,27,28].

### 2.3. CHA_2_DS_2_;-VASc Score for Atrial Fibrillation Stroke Risk

The CHA_2_DS_2_-VASc score has been validated by many studies, as it is used to evaluate the stroke risk in patients with AF [17]. The score administers 0 for the absence and 1 to 2 points for the presence of the following risk factors: hypertension 0 or 1, diabetes mellitus 0 or 1, age 75 years or older 0 or 2, congestive heart failure 0 or 1, vascular disease 0 or 1, stroke/thromboembolism 0 or 2, sex (female gender confers higher risk), and age 65 to 74 years 0 or 1. A score over 1 in males and over 2 in females recommends the initiation of anticoagulant therapy, and a score higher than 3 makes it mandatory to initiate this therapy due to an increased risk of stroke and other thromboembolic events [5].

### 2.4. Systematic COronary Risk Evaluation (SCORE2)

SCORE2 is a risk prediction algorithm for estimating the 10-year risk of fatal and non-fatal CV events in people aged 40–69 years from four geographic risk regions in Europe, facilitating the identification of individuals at risk of developing CVD. SCORE2 takes into consideration age, sex, smoking, SBP (mmHg), total cholesterol, and high-density cholesterol (HDL) levels (mg/dL). Romania, compared to other European regions, is considered one with very high risk. SCORE2-OP allows the estimation of cardiovascular event risk in people over 70 years old, and it is also employed for patients with DM-2 [37,38,39]. The European Society of Cardiology (ESC) the CVD Risk Calculation application was used for the patients included in our study to evaluate individual cardiovascular risk [38]. With the ESC CVD Risk Calculation App, SCORE2, SCORE2-OP, and SCORE2-DM were calculated. This application is powered by the ESC, based on the source codes of the U-Prevent web tool, a concept developed by the University Medical Center Utrecht and redesigned and owned by ORTEC [37,38,39].

### 2.5. Statistical Analysis

The statistical analysis was performed using IBM SPSS Statistics program software for Windows version 20.0 with a significance level of 0.05. Results are expressed as percentages for categorical data and as a mean value ± standard deviation for continuous data. We analyzed our patients based on four groups: patients with DM-2, patients with DM-2 and AF, patients with AF, and controls. Statistical analysis was based on unpaired *t*-tests for continuous variables and chi-square tests for categorical variables. In all patient groups, the assessment of blood tests, SBP, DBP, HR, LV function parameters, ABI, IMT, memory, activity, IADL, and depression tests was carried out using an impaired *t*-test. The chi-square test was used to examine differences between categorical variables, with the corresponding *p*-values presented in the summary tables. Correlations between key data were highlighted using the Spearman′s correlation coefficient. Bonnett and Wright’s method was used to estimate the corresponding 95% confidence intervals (95% CI) for the correlation coefficients [40].

Since a key outcome of our study was the MMSE values, we checked the corresponding achieved power for the MMSE values by using G* Power 3.1.9.2 software. We determined the achieved power for the corresponding comparisons of our four subgroups of patients and obtained the following results: in group A versus group B, we registered an effect size of 1.46 for corresponding sample sizes, and G* Power 3.1.9.2 software showed an achieved power of 100%. For group A versus group C, the effect size was 0.61, and the calculated achieved power was 92.06%; for group B versus group C, the effect size was −0.85, and the calculated achieved power was 99.63%; for group A versus group D, the effect size was −1.35, and the calculated achieved power was 100%; for group B versus group D, the effect size was −2.81, and the calculated achieved power was 100%, while for group C versus group D, the effect size was −1.96, and the achieved power was 100%. This shows that our sample size was large enough to draw statistically relevant conclusions, as a power of over 80% was achieved.

To evaluate the prognostic factors of CD with GDS-15, ADL, Score-2, age SBP, and DBP in DM-2 and AF patients, we used the statistical method of logistic regression, considering the forward method based on the Wald test to determine the significant variables for which the odds ratios (OR) with corresponding 95% confidence intervals (CI) were reported. In addition, for values of SCORE2, the final models were evaluated for sensitivity, specificity, positive predicted value (PPV), and negative predicted value (NPV), and the associated ROC curve with the corresponding area under the ROC curve were calculated. To determine goodness of fit, we employed Nagelkerke R-square for our final regression models. No adjustment for multiple comparison correction was considered since our study was a post hoc analysis of existing data, and the obtained results should be considered in conjunction with those from similar prospective studies for their validation [41].

## 3. Results

### 3.1. Characteristics of the Study Population

All 248 VHCVR patients enrolled in our study had a mean age of 71.42 ± 9.67 (45–89 years). Among them, 135 (54.40%) were females and 113 (45.60%) men. Overall, 74 (29.80%) patients were younger than 65 years old and 174 (70.20%) older than 65 years. Of all 248 VHCVR patients, 63 (25.40%) had DM-2 (group A), 60 (24.20%) had DM-2 and AF (group B), 61(24.60%) had AF (group C), and 64 (25.80%) did not have DM-2 and AF, comprising the control (group D). Of all 123 patients with DM-2, 60 (48.79%) also had AF (group B). Of all 121 patients with AF, 60 (49.58%) also had DM-2 (group B) (see Table 1).

Of all 248 included patients, 224 (90.30%) had SH, 207 (83.50%) had CHF, and 152 (61.30%) had CKD classified according to the estimated glomerular filtration rate (eGFR) as CKD stage 1 (eGFR > 90 mL/min/1.73 m^2^), stage 2 (eGFR between 60–89 mL/min/1.73 m^2^), stage 3 (eGFR 30–59 mL/min/1.73 m^2^), or stage 4 (eGFR between 15–30 mL/min/1.73 m^2^). In total, 138 (55.60%) had hyperlipemia, 76 (30.60%) were smokers, and 82 (33.10%) were diagnosed with obesity defined in concordance with body mass index (BMI) as grade 1 (BMI between 30–34,9 kg/m^2^), grade 2 (BMI between 35–39.9 kg/m^2^), or grade 3 (BMI over 40 kg/m^2^) (see Table 1).

Patients from group A had a mean age of 67.97 ± 10.39; those from group B had a mean age of 73.03 ± 9.05; in group C, the mean age was 72.21 ± 8.24; and in group D, the mean age was 68.67 ± 10.01 (see Table 2).

In group A, 57 (90.50%) had SH, 23 (36.50%) were smokers, 28 (44.40%) had hyperlipemia, and 38 (60.30%) had CKD (see Table 1). In group B, 56 (93.30%) had SH, 16 (26.70%) were smokers, 37 (61.10%) had hyperlipemia, and 43 (71.70%) had CKD (see Table 1). In group C, 56 (91.80%) had SH, 11 (18.00%) were smokers, 38 (62.30%) had hyperlipemia, and 38 (62.30%) had CKD. In group D, 55 (85.90%) had SH, 26 (40.60%) were smokers, 35 (54.70%) had hyperlipemia, and 33 (51.60%) had CKD (see Table 1).

Out of the 55 (85.90%) patients with SH from group D, 28 (43.70%) had SH grade I, 18 (28.10%) had SH grade II, and 9 (14.10%) SH grade III. Of the 56 (91.80%) hypertensive patients from group C, 7 (11.50%) had SH grade I, 20 (32.80%) had SH grade II, and 29 (47.50%) grade III. Of the 56 (93.30%) patients with SH of group B, 10 (16.70%) had SH grade I, 23 (38.30%) grade II, and 23 (38.30%) grade III. Of the 57 (90.50%) hypertensive patients in group A, 21 (33.30%) had SH grade I, 25 (39.70%) grade II, and 11 (17.50%) had grade III.

In group B, permanent AF prevailed, being diagnosed in 25 (41.67%) patients, followed by paroxysmal AF in 22 (36.67%) and persistent AF in 13 (21.54%). Of 61 patients with AF (group C), 32 (52.50%) patients had paroxysm AF, 14 (23.00%) had persistent AF, and 15 (24.60%) patients had permanent AF.

### 3.2. Comparative Analysis of Clinical and Biological Parameters in All Four Patient Groups

Regarding the hemodynamic parameters, DBP (mmHg) was statistically significantly higher in patients with DM-2 and AF (group B) (80.50 ± 14.94) compared to DBP (mmHg) (74.22 ± 15.22) in CVRFs control patients without DM-2 and without AF (group D) (*p* < 0.05), and also, the DBP (mmHg) of 79.52 ± 12.91 was statistically significantly higher in CVRFs patients with DM-2 (group A) compared to CVRFs control patients without DM-2 and without AF (group D) (74.22 ± 15.22, *p* < 0.05) (Table 2).

The values of SBP were higher in all three groups of patients (A, B, and C) versus control patients (group D) but not statistically significant (see Table 2).

Our findings revealed that BP values were higher in patients with both DM-2 and AF versus subjects suffering only from DM-2 or AF and that the lowest values of SH were encountered in patients with VHCVR but without one of the two main pathologies studied in this research. In patients with DM-2 and AF, the elevated values of HR, SBP, and DBP may be caused by a longer history of AF and/or SH (Table 2).

Regarding SH, we noticed that although SBP and DPB values were higher in group B versus patients from groups A or C, SBP values were higher in group A compared to those from group C, and DBP values were increased in group C versus group A, we did not find statistically significant differences between the four groups (see Table 2).

HR was statistically significantly higher in groups A, B, or C versus patients from group D (*p* < 0.05). Although the values of HR were higher in subjects from group B compared to those from groups A or C, and these values were more increased in DM-2 patients versus those with A alone, the differences were not statistically significant (see Table 2).

BMI and WC values were statistically significantly higher in groups A or B versus controls (*p* < 0.05), and there was a statistically significant difference regarding WC between groups group B and C (*p* < 0.05) (see Table 2).

The mean age was statistically significantly higher in groups B or C compared to group D (*p* < 0.05) and in patients with AF, in groups B or C, in comparison to those diagnosed only with DM-2, in group A (*p* < 0.005) (see Table 2).

Our results demonstrated that the female gender prevailed in patients with AF compared to those with DM-2, while for the male gender, the opposite was sustained (*p* < 0.05) (see Table 2).

The SCORE2 for our entire study group was 37.89 ± 14.16, with a minimum of 12 and a maximum of 65. The values of SCORE2 43.12 ± 12.46 from group B were statistically significantly higher compared to those from group D (*p* < 0.001). SCORE2 levels in groups A and C were statistically significantly higher compared to group D (*p* < 0.05) (see Table 2).

Also, SCORE2 values were higher in AF and DM-2 patients (group B) versus DM-2 patients (group A) or AF patients (group C), and the differences were not statistically significant.

The levels of high-density lipoprotein (HDL) cholesterol were statistically significantly lower in group B than in group D (*p* < 0.05) and statistically significantly higher in DM-2 patients (group A) versus patients with AF with/without DM-2 (groups B and C) (*p* < 0.05), suggesting that patients with AF are more compliant to lipid-lowering therapy than ones with only diabetes (see Table 2). Probably due to better control of the lipid profile through medication and diet, all patients with AF and/or DM-2 had lower but not statistically significant values of total cholesterol, low-density lipoprotein (LDL) cholesterol, and triglycerides (TG) compared to those without any of these pathologies and who were not treated with statins (see Table 2).

The eGFR values were statistically significantly lower in patients from group B (53.86 ± 14.71 mL/min), compared to those from group D (63.23 ± 19.26, *p* < 0.05). The eGFR levels were statistically significantly lower in patients from group A (53.98 ± 13.43) compared to those from group D (63.23 ± 19.26, *p* < 0.05) (see Table 2).

Urid acid values were significantly higher at 6.43 ± 1.30 in group B versus 5.89 ± 1.63 in group A (*p* < 0.05) (see Table 2).

### 3.3. IMT and ABI in All Four Groups of Patients

IMT measured on left CCA, 0.77 ± 0.26, in group B (patients with DM-2 and AF) was statistically significantly higher compared to the values determined in VHVR patients (group D—without DM-2 or AF) (*p* < 0.05). ABI values assessed in groups A and B were statistically significantly lower compared to group D (*p* < 0.05) (see Table 3).

These higher values of IMT and the lower ones of ABI in groups A, B, and C versus group D highlight that signs of subclinical atherosclerosis are more advanced in patients with DM-2 and/or AF compared to those without any of these two diseases (Table 3).

### 3.4. Echocardiographic Parameters of LV Systolic and Diastolic Function in Patient Groups

LVEF was significantly lower (55.61 ± 7.33) in group B compared to group D (58.35 ± 5.55, *p* < 0.05). Although LVEF was lower in groups A and C, compared to group D, the difference was not statistically significant (see Table 4).

The LA diameter was significantly higher (42.98 ± 6.89) in group B compared to group D (*p* < 0.05) (see Table 4).

Left ventricular end-systolic volume (LVESV) (ml) was significantly higher (40.14± 13.79) in group C compared to 33.75 ± 11.86 in group D or to 33.79 ± 14.92 in group A (*p* < 0.05) (see Table 4).

Echocardiographic parameters characterizing the right-heart function, estimated systolic pulmonary arterial pressure (sPAP), and the maximal velocity of the tricuspid regurgitation (TRVmax) were significantly higher in group B compared to group D or to group A (*p* < 0.05). These two parameters had significantly more elevated values in group C compared to group A (*p* < 0.05), and sPAP (mmHg) was significantly increased in group C compared to group D (*p* < 0.05) (see Table 4).

### 3.5. Comparative Analysis of Neuropsychological Tests in All Four Groups of Patients

From all 248 HCVRF patients evaluated in this study, 167 (67.3%) were patients with MMSE < 27; 32 (12.9%) patients had MMSE between 24 and 27, signifying a slight CD; and 49 (19.9%) individuals had values under 24, indicating dementia. The analysis of the results of the neuropsychological tests showed a statistically significant decrease in cognitive parameters (MMSE and MoCA) as well as in parameters assessing the patients’ daily activity (ADL and IADL) (*p* < 0.05) and a significant increase in markers of depression (GDS-15) between subjects from group B compared to those from group D (*p* < 0.05) (see Table 5).

MMSE and MoCA were statistically significantly lower in group B compared to group D (*p* < 0.05). MoCA was statistically significantly lower in patients with DM-2 and AF than in those without DM-2 and AF (*p* < 0.05). ADL and IADL were statistically significantly lower in patients with DM-2 and AF from group B compared to patients without DM-2 and AF from group D (*p* < 0.05). GDS-15 was statistically significantly higher in group B than in group D (*p* < 0.05) (see Table 5). The number of patients with MMSE between 24 and 27 was statistically significantly higher in groups B and C than in group D (*p* = 0.001). The number of patients with MMSE < 24 was statistically significantly higher in groups B and C than in group D (*p* = 0.001).

MMSE and ADL were statistically significantly lower and GDS-15 statistically significantly higher in patients with AF (group C) versus group D, while MoCA and IADL were statistically significantly lower in patients with DM-2 (group A) versus group D (*p* < 0.05) (see Table 5). The values of MMSE, MoCA, ADL, and IADL were lower, and those of GDS-15 were higher in patients with DM-2 and AF (group B) versus patients with only DM-2 (group A) or those only with AF (group C), but there were no statistically significant differences. The values of MMSE and ADL were lower and GDS-15 higher in patients with AF (group C) versus patients with DM-2 (group A). The levels of MoCA and IADL were lower in patients with DM-2 (group A) versus those with AF (group C), but no statistically significant differences were obtained (see Table 5).

Considering SCORE2 classification, of all 248 VHCVR patients included in this study, SCORE2 was statistically significantly higher in patients with MMSE < 24 versus those with MMSE between 24 and 27 (*p* < 0.05) and in subjects with dementia compared to those with MMSE > 27 (*p* < 0.05).

When analyzing only 184 patients with DM-2 and/or AF, SCORE2 was statistically significantly higher in subjects with MMSE < 24 versus those with MMSE between 24 and 27 (*p* < 0.05) and patients with MMSE > 27 (*p* < 0.05).

In patients with MMSE < 24, SCORE2 was statistically significantly higher in group B (46.98 ± 11.88) versus 36.02 ± 8.20 in group D (*p* < 0.05). In patients with MMSE < 27, SCORE2 was statistically significantly higher: 45.52 ± 12.23 in group B (DM-2 and AF patients) versus 34.46 ±13.32 in group D (patients without AF and DM-2) (*p* < 0.05).

In patients with MMSE between 24 and 27, SCORE2 was the highest in those with DM-2 and AF (45.52 ± 12.23), followed by patients diagnosed only with AF (41.84 ± 12.24) and by those with DM-2 (39.10 ± 12.94), and the lowest values were determined as 34.46 ± 13.32 in patients with VHCVR but without AF or DM-2.

In patients with MMSE < 24, SCORE2 was the highest in patients with both DM-2 and AF (46.98 ± 11.98), and it was 43.84 ± 9.09 in subjects with DM-2 and 43.23 ± 11.98 in those with AF and 36.02 ± 8.20 in group D.

Our results demonstrated that patients with associated DM-2 and AF may have a slightly altered daily activity and a greater degree of cognitive impairment and depression compared to patients diagnosed with only one of these two pathologies. The patients from group D, with neither DM-2 nor AF, although at VHCVR, had the lowest levels of CD or depression and the highest scores for the evaluation of functional status.

### 3.6. Correlation Between SCORE2, LVEF, Age, SBP, DBP, NT Pro-BNP Levels, and MMSE, MoCA, ADL, IADL, and GDS-15 and Correlation Between SCORE2 and NT Pro-BNP Levels in Patients with DM-2 and/or AF and CVRFs Patients

We determined a moderate positive statistically significant correlation between LVEF and ADL and IADL (*p* ˂ 0.001) and a moderate statistically significant but negative correlation with GDS-15 (*p* ˂ 0.001) (see Table 6).

In our study group, NT pro-BNP levels were correlated in a weak negatively statistically significant way with MMSE (r = −0.179, 95% CI −0.298; −0.055, *p* = 0.005), MoCA (r = −0.125, 95% CI −0.246; −0.001, *p* = 0.049), ADL (r = −0.176, 95% CI −0.295; −0.054, *p* = 0.005), and IADL (r = −0.128, 95% CI −0.249; −0.003, *p* = 0.044) (see Table 6).

In our study group, regarding SBP values, we noticed a moderate negative statistically significant correlation with MMSE, MoCA, and IADL (*p* < 0.001); a weak negative statistically significant correlation with ADL (*p* = 0.006); and a moderately positive one with GDS-15 (*p* < 0.001). Regarding DPB, we noticed a moderate negative statistically significant correlation with MMSE, MoCA, ADL, and IADL (*p* < 0.001) and a moderate positive one with GDS-15 (*p* < 0.001) (see Table 6).

We determined that the age had a low negative statistically significant correlation with MoCA (*p* = 0.005); a moderate one with MMSE, ADL, and IADL (*p* < 0.001); and a statistically moderate positive correlation with GDS-15 (*p* ˂ 0.001) (Table 6).

Regarding SCORE2 values, the correlation was weakly negative but statistically significant with MMSE (r = −0.200, 95% CI −0.318; −0.076, *p* = 0.002), MoCA (r = −0.180, 95% CI −0.299; −0.056, *p* = 0.004), ADL (r = −0.161, 95% CI −0.281; −0.036, *p* = 0.011), and IADL (r = −0.126, 95% CI −0.247; −0.001, *p* = 0.048) and weakly positively statistically significant with GDS-15 (r = 0.160, 95% CI 0.035; 0.280, *p* = 0.011) (see Table 6).

We documented low-positive but statistically significant correlations between SCORE2 values and NT pro-BNP levels (r = 0.138, 95% CI 0.004; 0.257 *p* = 0.029).

### 3.7. Evaluation of Prognostic Factors on CD Depending on CVRFs, DM-2, and AF

To evaluate the prognostic factors for developing CD, as quantified by MMSE between 24 and 27, and dementia (MMSE < 24) in the entire study group of 248 VHCVR patients and in 184 patients with DM-2 and/or AF (groups A, B, and C) and to assess the odds ratio, we built a logistic regression model for these subjects to identify the most important predicting factors and SCORE2, and a similar model was also created for age, SBP, and DBP. We used a similar model to identify the impact of ADL and GDS-15. A forward method based on the Wald test was employed to determine the significant variables for SCORE2. The model was evaluated through sensitivity, specificity, PPV, NPV, and the associated ROC curve with the corresponding area under the ROC curve. Logistic regression analysis revealed statistically significantly higher odds of CD with higher SCORE2 values when considering all 248 patients or patients with AF and/or DM-2.

The odds of MMSE < 27 were higher with increased SCORE2. In these patients, for an additional 1% in SCORE2 values, the odds of CD and MMSE between 24 and 27 were higher by a factor of 1.024 (OR = 1.024, 95% CI 1.001; 1.048) in AF and/or DM-2 patients and were higher by a factor of 1.031 (OR = 1.031, 95% CI 1.011; 1.052) in all patients (see Table 7). In other words, an increase of 1% in SCORE2 was associated with an increase of 2.4% in the odds of CD (MMSE < 27) in DM-2 and/or AF patients. This means that if new elements of cardiovascular risk appear (the SCORE2 increased by 1%), the risk of cognitive impairment increases with an increase of 2.4% in the odds of MMSE between 24 and 27. Also, an increase of 1% in SCORE2 was associated with a 3.1% increase in the odds of CD in all patients with VHCVR. This means that if new elements of CVRF develop (the SCORE2 increased with 1%), the risk of cognitive impairment increases (with an augmentation of 3.1% in the odds of MMSE between 24 and 27).

**Figure 1:**
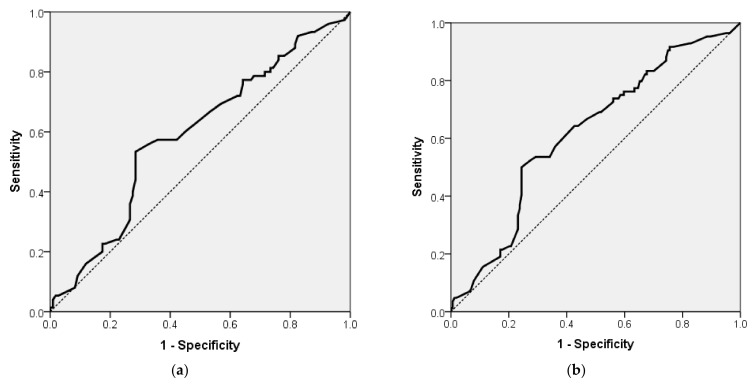
(**a**) Receiver operating characteristic (ROC) curve for multiple logistic regression model for the association of MMSE < 27 for patients with DM-2 and/or AF with SCORE2 (AUROC = 0.589, 95% CI 0.505; 0.673, *p* = 0.041). (**b**) Receiver operating characteristic (ROC) curve for multiple logistic regression model for the association of MMSE < 27 for all VHCVR patients with SCORE2 (AUROC = 0.619, 95% CI 0.546; 0.692, *p* = 0.002).

The probability of MMSE < 27 for patients with DM-2 and/or AF can be estimated by the following formula: exp (RS)/(1 + exp (RS)), where RS = −1.347 + 0.024 × (SCORE2). This model classified 59.78% of the patients correctly. The model had a sensitivity of 5.33%, a specificity of 97.25%, a PPV of 57.14%, and an NPV of 59.89%. The area under the ROC curve for this model (Figure 1a) was 0.589 (AUROC = 0.589, 95% CI 0.505; 0.673, *p* = 0.041).

The probability of MMSE < 27 for all VHCVR patients can be estimated by the following formula: exp (RS)/(1 + exp (RS)), where RS = −1.875 + 0.031 × (SCORE2). This model classified 66.13% of the patients correctly. The model had a sensitivity of 1.19%, a specificity of 99.39%, a PPV of 50.00%, and an NPV of 66.26%. The area under the ROC curve for this model (Figure 1b) was 0.619 (AUROC = 0.619, 95% CI 0.546; 0.692, *p* = 0.002).

Logistic regression analysis revealed statistically significantly higher odds of CD with increased age and higher SBP and DBP values when considering all patients with VHCVR, namely patients with AF and/or DM-2.

In these patients, for an additional year in age, the odds of CD, i.e., MMSE between 24 and 27, were higher by a factor of 1.123 (OR = 1.123, 95% CI 1.076; 1.173) in all patients and by a factor of 1.140 (OR = 1.140, 95% CI 1.081; 1.201) in AF and or DM-2 patients (see Table 8). For an additional 1 mmHg in SBP, the odds of MMSE between 24 and 27 were higher by a factor of 1.025 (OR = 1.025, 95% CI 1.005; 1.046) in all patients and by a factor of 1.023 (OR = 1.023, 95% CI 1.001; 1.046) in DM-2 and/or AF patients. For an additional 1 mmHg in DBP, the odds of MMSE between 24 and 27 were higher by a factor of 1.052 (OR = 1.052, 95% CI 1.022; 1.084) in all patients and by a factor of 1.080 (OR = 1.080, 95% CI 1.039; 1.123) in DM-2 and/or AF patients. The corresponding Nagelkerke R-square of the final models for all patients was 0.419 and 0.486 for the DM-2 and/or AF group.

Our logistic regression analysis revealed statistically significantly higher odds of MMSE between 24 and 27 and of dementia (MMSE < 24) for increased GDS-15 score and statistically significantly lower odds of MMSE between 24 and 27 and also of dementia (MMSE < 24) for increased ADL score when considering all 184 patients with DM-2 and/or AF or all 248 subjects (see Table 9).

Logistic regression analysis revealed statistically significantly higher odds of CD in patients with DM-2 and/or AF. For an additional 1 unit in GDS-15 score, the odds of CD were higher by a factor of 1.363 (OR = 1.363, 95% CI 1.142; 1.627) (*p* < 0.05). In other words, an increase of 1 unit in GDS-15 was associated with an increase of 36.3% in the odds of developing MMSE < 27. We determined statistically significantly lower odds of MMSE between 24 and 27 for an additional unit of ADL score, with the odds of CD being lower by a factor of 0.560 (OR = 0.560; 95% CI = 0.396; 0.790) (*p* < 0.05). In other words, an increase of 1 unit in ADL score was associated with a 44.0% decrease in the odds of CD. This means that if new elements of depression occur (the GDS score increased with 1 unit), the risk of cognitive impairment increases (with an increase of 36.3% in the odds of MMSE between 24 and 27), and if the quality of life is improved (the ADL score increased with 1 unit), the risk of CD decreases (with a 44.0% decrease in the odds of MMSE < 27). The corresponding Nagelkerke R-square of this final model was 0.290.

Logistic regression analysis revealed statistically significantly higher odds of CD in patients all VHCVR patients. For an additional 1 unit in GDS-15 score, the odds of CD were higher by a factor of 1.374 (OR = 1.374, 95% CI 1.176; 1.606) (*p* < 0.05). In other words, an increase of 1 unit in GDS-15 was associated with an increase of 37.4% in the odds of MMSE < 27. We determined statistically significantly lower odds of MMSE between 24 and 27 for an additional unit of ADL score, with the odds of CD being lower by a factor of 0.485 (OR = 0.485; 95% CI = 0.352; 0.667) (*p* < 0.05). In other words, an increase of 1 unit in ADL score was associated with a 51.5% decrease in the odds of CD. The corresponding Nagelkerke R-square of this final model was 0.329.

Referring to MMSE under 24, signifying dementia, the logistic regression analysis revealed statistically significantly higher odds for MMSE to decrease under 24 for an increase in SCORE2 (%) (see Table 10), age, and DBP (see Table 11). SBP had no statistical significance when considered in the corresponding models with age and DBP.

Logistic regression analysis revealed statistically significantly higher odds of MMSE < 24 (dementia) for increased SCORE2 when considering patients with DM-2 and/or AF and the entire study group.

The odds for developing MMSE < 24 (dementia) were higher for increased SCORE2. For an additional unit in SCORE2, the odds of MMSE < 24 (dementia) were higher by a factor of 1.043 (OR = 1.043, 95% CI 1.013; 1.074) in patients with DM-2 and/or AF. In other words, an increase of 1% in SCORE2 was associated with a rise of 4.3% in the odds of MMSE < 24 (dementia) in patients with DM-2 and/or AF.

For an additional unit in SCORE2, the odds of MMSE < 24 (dementia) were higher by a factor of 1.047 (OR = 1.047, 95% CI 1.021; 1.074) in all VHCVR patients. In other words, an increase of 1% in SCORE2 was associated with an increase of 4.7% in the odds of MMSE < 24 (dementia) in all patients.

**Figure 2:**
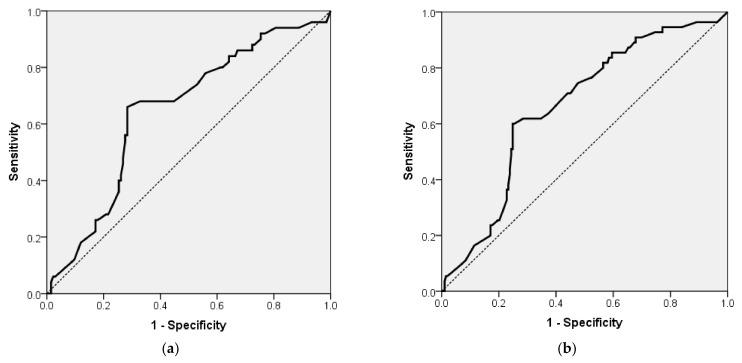
(**a**) Receiver operating characteristic (ROC) curve for multiple logistic regression model for the association of MMSE < 24 for patients with DM-2 and/or AF with SCORE2 (AUROC = 0.646, 95% CI 0.558; 0.734, *p* = 0.002); (**b**) receiver operating characteristic (ROC) curve for multiple logistic regression model for the association of MMSE < 24 for all VHCVR patients with SCORE2 (AUROC = 0.662, 95% CI 0.585; 0.739, *p* < 0.001).

The probability of MMSE < 24 (dementia) for patients with DM-2 and/or AF can be estimated by the following formula: exp (RS)/(1 + exp (RS)), where RS = −2.753 + 0.042 × (SCORE2). This model classified 72.83% of the patients correctly. The area under the ROC curve for this model (Figure 2a) was 0.646 (AUROC = 0.646, 95% CI 0.558; 0.734, *p* = 0.002).

The probability of MMSE < 24 (dementia) for all VHCVR patients can be estimated by the following formula: exp (RS)/(1 + exp (RS)), where RS = −3.116 + 0.046 × (SCORE2). This model classified 77.82% of the patients correctly. The area under the ROC curve for this model (Figure 2b) was 0.662 (AUROC = 0.662, 95% CI 0.585; 0.739, *p* < 0.001).

The statistical regression analyses revealed that in all patients and those with DM-2 and/or AF, the odds of dementia, characterized by a decrease in MMSE under 24, were higher for older patients and those with higher DBP. The corresponding Nagelkerke R-square of the final models for all patients was 0.386 and 0.409 for the DM-2 and/or AF patients, respectively.

For an additional increase of one year in age, the odds of dementia were higher by a factor of 1.154 (OR = 1.154, 95% CI 1.096; 1.214) in patients with VHCVR (*p* < 0.001). For any additional increase of 1 mmHg in DBP, the odds of dementia were higher by a factor of 1.077 (OR = 1.077, 95% CI 1.048; 1.106) (*p* < 0.001).

For an additional increase of one year in age, the odds of dementia were higher by a factor of 1.147 (OR = 1.147, 95% CI 1.083; 1.214) in DM-2 and/or AF patients (*p* < 0.001). For any additional increase of 1 mmHg in DBP, the odds of dementia were higher by a factor of 1.095 (OR = 1.095, 95% CI 1.058; 1.133) in DM-2 and/or AF patients (*p* < 0.001). SBP was not statistically significant.

Referring to dementia, the logistic regression analysis revealed statistically significantly higher odds of MMSE < 24 for an increase in the severity of depression (GDS-15 score) and statistically significantly lower odds of MMSE < 24 for an increase in the activity of daily living (ADL score) when considering all VHCVR patients and DM-2 and/or AF patients (*p* < 0.05) (see Table 12).

For an additional 1 unit in GDS-15 score, the odds of dementia were higher by a factor of 1.521 (OR = 1.521, 95% CI 1.256; 1.841) (*p* < 0.001) in all patients. In other words, an increase of 1 unit in GDS-15 was associated with an increase of 52.1% in the odds of MMSE < 24.

We observed statistically significantly lower odds of MMSE < 24 for an additional unit of ADL score. The odds of dementia were lower by a factor of 0.508 (OR = 0.508; 95% CI = 0.372; 0.694; (*p* < 0.001) in all patients. In other words, an increase of 1 unit in ADL score was associated with a 49.2% decrease in the odds of MMSE < 24. This means that if new elements of depression occur (the GDS-15 score increased with 1 unit), the risk of dementia (MMSE < 24) increases (with an increase of 52.1% in the odds of MMSE < 24), and if the quality of life is improved (the ADL score increased with 1 unit), the risk of dementia decreases (with a 49.2% decrease in the odds of MMSE < 24). The corresponding Nagelkerke R-square of this final model was 0.377.

We observed statistically significantly lower odds of MMSE < 24 for an additional unit of ADL score. The odds of dementia were lower by a factor of 0.634 (OR = 0.634; 95% CI = 0.454; 0.884; (*p* = 0.007). This means that if new elements of depression occur (the GDS-15 score increased with 1 unit), the risk of dementia (MMSE < 24) increases (with an increase of 57.4% in the odds of MMSE < 24), and if the quality of life is improved (the ADL score increased with 1 unit), the risk of dementia (MMSE < 24) decreases (with a 36.6% decrease in the odds of MMSE < 24). The corresponding Nagelkerke R-square of this final model was 0.336.

## 4. Discussion

The impact of CVD and DM-2 in individuals with a VHCVR profile, as assessed by SCORE2, that may trigger and amplify various neuropathological mechanisms and thereby initiate the development of CD by progressively reducing the brain’s functional reserve is a largely debated topic in the medical literature [1]. However, other important contributing factors, such as the socioeconomic status and medication adherence in both DM and AF patients, play an important role, severely altering those patients’ mental health status and quality of life, as presented in several studies [42,43,44]. Factors associated with low adherence to treatment included low income, age <50 years or >80 years), low socioeconomic status, unemployment, food and/or job insecurity, medication costs, lack of information and medical knowledge related to one’s disease and the medication needed to treat it, beliefs regarding medication, patient motivation to make necessary behavioral changes, problems related to the health system, and logistical and social barriers [42,43,44].

DM-2 is thought to favor the early onset of systemic atherosclerosis [2,45], as it is associated with a 1.5-fold increased risk of developing CD and dementia, with more than 45% of diabetic patients showing mild signs of CD [1,2,46]. The number of people suffering from DM-2 is rapidly increasing globally, doubling in the last thirty years and rendering it a major public health challenge [45]. Approximately 90% have DM-2, and there is a close relationship between DM-2, age, and CD [45]. Subclinical and clinical atherosclerosis and the neurodegenerative effects of hypo- and hyperglycemia favor the early onset of DC [2,22]. Several specialized literature studies have highlighted that people with DM-2 have a lower cognitive MMSE score than those without diabetes [47,48] and that there is a link between diabetes in middle age and the presence of greater CD [47,49].

In a study conducted on 1519 elderly patients ≥ 75 years old with DM-2, the changes in the MMSE score were evaluated. Patients were divided into groups based on glycosylated hemoglobin (HbA1) levels. Age, less physical activity, history of cerebrovascular disease, baseline MMSE score, and HBA1 ≥ 8% were associated with CD. This study concluded that in elderly patients with DM-2, HbA1 ≥ 8% is an independent factor for CD and is also associated with CD severity (*p* = 0.029) [47]. In our study in patients with DM-2 and AF (group B), MMSE was statistically significantly lower compared to nondiabetic patients (OR = 1.55, 95% CI: 1.13–2.12, *p* = 0.007).

In another study in Mysuru (India) that evaluated CD in patients with/without DM who were followed for 4 months, the MoCA evaluation scale was used. A significantly lower MoCA score was found in patients with DM 18.99+/−0.48 compared to those without DM (26.21+/−0.46, *p* < 0.001). This study highlighted that factors such as gender, education level, geographic location, smoking, DM duration, and regular medication use did not significantly influence the cognitive performance as assessed by MoCA in DM patients [13]. In our study, in patients with DM-2 (group A) and in patients with DM-2 and AF (group B), MoCA was statistically significantly lower compared to control patients (*p* < 0.005).

In a study that evaluated 2577 Swedish patients aged over 60 years without dementia for over 12 years, the presence of cognitive changes and the incidence of CD associated or not with dementia were analyzed. Cardiometabolic diseases (CMD) were defined as the presence of DM, CVD, and stroke [50]. Simultaneous CMDs were more frequent with the aging of the population [50,51]. As in our study, where we evaluated the presence of CD in patients with DM with or without AF, this study examined the impact of CMD on CD and its progression to dementia and concluded that CMD multimorbidity accelerates CD and increases its risk of conversion to dementia by approximately 2 years [51]. Since several studies have reported associations between CMD, CVDRF, and CD, the need to reconsider the impact of various CVRFs on the development of CD and dementia is to be considered [50,52,53]. Poor glycemic control (HbA1c ≥ 7.0% to 7.5%) was also associated with an increased risk of CD [50]. Better control of CVDRF could allow a better protection of cognition [50]. In our study, in patients with AF and in those with DM-2 and AF, MMSE was statistically significantly lower compared to control patients (*p* < 0.05).

Some studies have shown that the relationship between AF and Alzheimer’s dementia is independent of the incidence of stroke [54]. The increase in the number of elderly patients suffering from AF also has an important impact on the development of CD, affecting their quality of life [54,55]. Recent studies have reported that a history of AF, CCS, and CHF is associated with a 77% increased risk of mild CD [56]. The detrimental impact of CVD on cognition may be explained by a reduction in cerebral blood flow and oxygen supply or disruption of the blood–brain barrier [50,57], which can lead to neurological damage such as cerebral infarctions and an increased accumulation of white matter lesions [50,57,58]. Regarding the TTE parameters in our study, LVEF was significantly lower and LA dimension higher in DM-2 and AF patients compared to controls (*p* < 0.05). Also, LVESD was significantly lower in DM-2 patients, and LVESV was significantly higher in AF patients compared to VHCVR patients without AF and DM-2 (*p* < 0.05). Regarding LVEF, in patients with DM-2 and/or AF and CVRFs, we noticed a moderate positive statistically significant correlation with ADL and IADL (*p* < 0.001) and a significant moderate negative correlation with GDS-15 (*p* < 0.001).

Recent data suggest that AF confers a higher risk for developing early-onset dementia, regardless of clinical stroke. Numerous mechanisms have been advanced to explain CD in AF, including silent cerebral infarctions (SCI), cerebral hypoperfusion, and cerebral microvascular disease [6]. Several studies have shown that SCI is associated with future clinical stroke and dementia [7]. In a meta-analysis, AF was associated with a 2.6-fold increased risk of SCI [7,59]. Cerebral microembolization and hypoperfusion associated with AF can cause cerebral ischemic demyelination like that seen in cerebral small vessel disease (CSVD), which may favor CD. AF is often associated with an increased prevalence of CSVD, which may be a marker for cognitive changes [7,60]. In a study that assessed the presence of SCI by magnetic resonance imaging, patients with AF had lower memory scores than patients with sinus rhythm [7]. The results of the neuropsychological tests in our study showed a statistically significant decrease in cognitive parameters (MMSE and MoCA) (*p* < 0.05) as well as in those assessing the daily activity (ADL and IADL) (*p* < 0.05) and a significant increase in the parameters characterizing depression (GDS-15) (*p* < 0.05) in group B compared to group D (*p* < 0.05). MMSE and ADL were statistically significantly lower and GDS-15 statistically significantly higher in group C versus group D, and MoCA and IADL were significantly lower in group A versus group D (*p* < 0.05). This suggests that patients with VHCVR and associated DM-2 and AF have more severe CD and a greater impairment of daily activity and depression compared to those with AF or without DM-2 and AF.

In the Atherosclerosis Risk in Communities (ARIC) study, which followed patients for 20 years, 2106 developed AF and 1157 dementia. A greater decrease in cognitive function was observed in participants with AF compared to those without AF. Of 935 patients without stroke, only in the subgroup with SCI was a decline in executive function and verbal fluency associated with the incidence of AF. This finding suggests that vascular damage is likely the cause of CD. SCI may be an intermediate stage of the association between AF and CD [7,61].

In a study evaluating patients with AF by Holter monitoring, those with lower (<50 bpm) or higher (>90 bpm) ventricular response rates had a 7-fold higher risk of dementia compared with patients with a moderate ventricular response rate. These high or low ventricular rates are causes of cerebral hypoperfusion [7,62]. In our study, HR was statistically significantly higher in groups A and B versus group D (*p* < 0.05).

In a study of 358 patients aged over 65 years with cognitive impairment (MMSE < 24) followed-up over 10 years, AF was associated with a 4-fold higher risk of dementia [7,63]. In our study, the statistical regression analysis revealed that in all patients with VHCVR and especially in those with associated DM-2 and/or AF, the odds of dementia were higher for older patients and those with higher DBP values.

In a Korean study, in an elderly population, the incidence of AF was associated with an increased risk of dementia independent of stroke; treatment with oral anticoagulants (OACs) and good BP control were linked to a lower incidence of dementia [64]. In our study, DBP values were statistically significantly higher in patients with DM-2 and AF (group B) compared to patients with VHCVR without DM-2 and AF (group D) (*p* < 0.05), and DBP values were statistically significantly higher in group A compared to group D (*p* < 0.05).

In a study evaluating 6514 participants without dementia, in younger participants, new-onset dementia was strongly associated with the duration of AF [65]. In the ARIC study, persistent AF and not paroxysmal AF were associated with lower cognitive function [61]. AF decreases cardiac output and BP [12]. Another study demonstrated that patients with persistent AF had hypoperfusion of the brain tissue compared to those with paroxysmal AF and in sinus rhythm [66]. Changes in HR may also contribute to impaired cerebral perfusion by decreasing cardiac performance [67]. In our study, of 60 patients with DM-2 and AF (group B), 41.70% had permanent AF, and of 61 patients with AF (group C), permanent AF was present in 24.60%. In patients with AF, there was a relationship between the CHA_2_DS_2_-VASc score and the risk of dementia, with higher scores being associated with an increased risk of dementia [68]. Thus, patients with higher CHA_2_DS_2_-VASc scores could best benefit from screening for CD and should be followed-up and selected for early interventions to prevent dementia. It is debated how to manage VHCVR subjects with AF and lower CHA_2_DS_2_-VASc scores to delay the onset of CD. Good management of AF and associated risk factors resulted in a lower risk of dementia [69]. In our study, the mean CHA_2_DS_2_-VASc score was 3.57 ± 1.35 in 121 patients with AF with or without DM-2 (groups B and C). Of all these subjects, 47 had CHA_2_DS_2_-VASc >3 (17 had CD, and 30 had dementia). The number of patients with MMSE < 24 and CHA_2_DS_2_-VASc > 3 was higher in group B than in group C. In our study, MMSE, MoCA, and ADL were statistically significantly lower and GDS-15 statistically higher in patients with CHA_2_DS_2_-VASc >3 than in patients with CHA_2_DS_2_-VASc < 3 (*p* < 0.05). In another study, similar results were obtained when comparing hypertensive patients with AF and CHA_2_DS_2_-VASc scores over and under 3, respectively [4].

Several studies suggest that inflammation is a nonspecific marker of atherosclerotic vascular disease that has been associated with both CD and AF [55,70]. Markers of subclinical CVD and atherosclerosis (aortic stiffness and IMT) have been associated with an increased risk of AF and CD [7,18,70]. In our study, higher values of IMT and lower values of ABI in DM-2 or AF patients or in those with both DM-2 and AF versus group D indicated that the process of subclinical atherosclerosis is more advanced in the first category of subjects. Inflammation is an important component of the pathophysiological process that could increase hypercoagulability and lead to the formation of potential thrombus, increasing the risk of stroke and cerebrovascular dysregulation, which has been associated with vascular and Alzheimer’s dementia [7,71]. Inflammation can induce AF, and in turn, AF exacerbates the inflammatory response [7].

It was considered that regular physical activity and management of comorbidities associated and CV risk factors (DM, SH, obesity, and smoking) could reduce the risk of CD [47,72]. In our study, BMI and WC were statistically significantly higher in group A and in group B versus group D (*p* < 0.05), and also, the values of WC were statistically significantly higher in group B versus group C (*p* < 0.05). This indicates that patients with DM-2 and with DM-2 and AF, respectively, have a higher BMI and WC compared to those with AF. Thus, more attention should be given to lifestyle changes, management of depression, and higher daily activity to postpone the onset of CD in patients with DM-2 and with associated DM-2 and AF.

The presence of SH both in young adults and in older people is an important risk factor for the development of CD but also of vascular and Alzheimer’s dementia. Both SH and dementia are common conditions in the general population [1]. SH and AF in VHCVR patients favors the onset of CD [4]. The better we control these pathologies, the more we can prevent stroke and dementia. [4] Moreover, in hypertensive patients with associated DM-2 and AF, increased attention is needed to detect early signs of CD. In our study, over 90% of patients from groups A, B, and C had SH as well as 85% of those with VHCVR but without DM-2 and AF. We determined a negative significant correlation between BP values and MMSE, MoCA, IADL, and ADL and a moderate positive one with GDS-15.

Age-related CD can be attributed to the reduced size of the hippocampus, which leads to neurodegeneration and impaired neuroplasticity [13,73]. Age was related to the severity of CD, expressed through MMSE values, in both populations with and without DM-2 [47]. Several studies have reported that in patients with DM-2, age has a significant influence on CD (poor MoCA score performers) (*p* < 0.001) [13,74]. We determined in our study that age had a mild negative statistically significant correlation with MoCA (*p* = 0.005) and moderate one with MMSE, ADL, and IADL (*p* < 0.001), and it was significantly moderately positively correlated with GDS-15 (*p* < 0.001).

Other studies have reported associations between SH, CCS, and DM-2 and low performance in cognition between patients with high Framingham CVD risk scores and low cognitive scores [50,75,76]. In these patients, CD developed earlier in people younger than 78 [48,71], indicating that patients with VHCVR can develop CD sooner. [50,75,76]. Our results show that age, SBP, DBP, SCORE 2 values, NT-pro-BNP, and LVEF values play an important role in the relationship between CD DM-2 and AF.

Patients with DM-2 are two to three times more likely to be diagnosed with depression compared to those without DM-2 [77,78]. In patients with DM-2, the presence of depression can lead to a worsening of the metabolism and HbA1c, with an increased risk of microvascular and macrovascular complications and deterioration of the quality of life. In one study, the presence of moderate depression was observed in 8–16% of patients with type 1 or type 2 DM [77,79]. In another study, depression was significantly associated with a nearly 1.25- to 2.24-fold increased risk of all-cause mortality in people with DM [77,80]. A meta-analysis evaluating 26 cross-sectional studies found a significant association of depression with hyperglycemia [77,81]. Fluctuations in BBG can determine a variety of symptoms, such as hunger, confusion, aggression, and irritability. Stress can lead to unhealthy eating behavior that affects glycemic control [77]. In DM-2 patients with or without depression, in a meta-analysis that evaluated the risk of CD and its progression to dementia, it was shown that in DM-2 patients with depression, the risk of CD and dementia is increased in comparison to DM-2 patients without depression [82]. In our study, GDS-15 was statistically significantly higher in patients with AF and in those with DM-2 and AF (*p* < 0.05). Depression is related to poor glycemic control, resulting in poor adherence to dietary regimens and medications, as it affects the patient’s psychosocial life, reducing the quality of life [77,83]. In another article that evaluated 104 patients with DM-2 and AF, it was found that MMSE, MoCA, ADL, and IADL were statistically significantly lower and GDS-15 significantly higher in patients with DM-2 and AF compared to controls without DM-2 or AF (*p* < 0.05) [2]. In another article that evaluated 155 patients with SH, in 84 of them with associated AF, MMSE, and MoCA were statistically significantly lower and GDS-15 significantly higher compared to controls without AF (*p* < 0.05) [4]. In our study, patients with DM-2 and AF as well as patients with AF had a statistically significantly lower ADL score than the controls (*p* < 0.05). The IADL score was statistically significantly lower in patients with DM-2 as well as in those with DM-2 and AF, highlighting that these patients have impaired daily activity and depressive symptoms, but when the two diseases were associated, their daily activity was more affected, and they had more elements of depression compared to patients without DM-2 or AF. Referring to CD, our logistic regression analysis revealed statistically significantly higher odds of MMSE between 24 and 27 or MMSE < 24 for an increase in depression (GDS-15 score) and statistically significantly lower odds of MMSE between 24 and 27 or MMSE < 24 for an increase in the activity of daily living (ADL score) when considering the entire study group as well as DM-2 and/or AF patients (*p* < 0.05). We determined that if new elements of depression occur (the GDS-15 score increased with 1 unit), the risk of dementia (MMSE < 24) increases by 52.1%, and if the quality of life is improved (the ADL score increased with 1 unit), the risk of dementia (MMSE < 24) decreases with 49.2% in the entire study group. In DM-2 and/or AF patients, the occurrence of elements of depression elevate the risk of dementia by 57.4%, and if the quality of life improves, the risk of dementia decreases by 36.6%.

Epidemiological studies suggest that approximately one-third of all cases of Alzheimer’s disease could be attributed to modifiable CV risk factors such as physical inactivity, obesity, smoking, SH, and DM [84].

It is vital to detect early signs of subclinical atherosclerosis for the strategy of primary prevention of CVD [85]. In patients with prediabetes and newly diagnosed DM-2 with associated CVD, carotid IMT evaluation is recommended as a routine screening [2]. In people with CD, carotid atherosclerosis in midlife has been associated with poorer cognitive performance, smaller brain volumes, and reduced cerebral blood flow [86]. In our study, left IMT was statistically significantly higher in group B compared to group D (*p* < 0.05). A study that, over 7 years, followed-up the IMT in 142 patients with DM-2 and CKD concluded that IMT is a strong independent predictor of CV morbidity and mortality [87]. In patients with leukemia and VHCVR, IMT was employed to assess vascular remodeling [88]. Similarly, in another study, the values of IMT, SBP, DBP, total cholesterol, LDL-cholesterol, triglycerides, and serum creatinine were higher in patients with DM-2 and prediabetes than in control patients [89]. In 3399 patients with DM-2, obesity and CD were independent risk factors for CKD progression, especially in women [90]. In our study, eGFR was statistically significantly lower in groups B and A compared to group D (*p* < 0.05).

One of the most important long-term complications of DM is PAD, and it is also suggested that there is a strong relationship between PAD and AF. One study determined that an ABI score < 1.00 indicated an increased risk of AF (HR = 1.32; 95% CI: 1.18–1.47). The presence of PAD was associated with a 31% increased risk for the incidence of AF [91]. In another study, from a group of 85 patients, 40 subjects with DM had lower ABI values compared to those without DM [92]. We had similar results in our study: subjects with DM-2 and/or AF had higher ABI levels compared to patients without DM-2 and AF.

The PESA (The Progression of Early Subclinical Atherosclerosis) study, which included 4184 asymptomatic individuals aged between 40 and 54 years, evaluated the presence of subclinical atherosclerosis and assessed cardiovascular risk using SCORE2 and brain structural changes by brain [^18^F] fluorodeoxyglucose ([^18^F] FDG)-PET. For cardiovascular risk stratification, patients were divided into three groups: low risk (SCORE2 < 2.5%), moderate risk (SCORE2 2.5–5%), and high risk (SCORE2 > 5%). The results showed that asymptomatic middle-aged individuals at high CVD risk have a greater decline in cerebral glucose metabolism over 5 years compared to low-risk individuals. In those at elevated risk, 20% of observed CD can be attributed to neurodegeneration and thus could be irreversible. An overall decrease of 1.5%, 3.1%, and 4.3% in cerebral metabolism was observed in the low-risk, moderate-risk, and high-risk groups, respectively. Patients at high cardiovascular risk had a three times greater average decrease in cerebral metabolism than low-risk individuals. This means that middle-aged people with persistently elevated cardiovascular risk had a 0.9% decrease in cerebral metabolism per year. Therefore, the observed changes in brain glucose metabolism could affect the brain’s ability to prevent neurodegenerative or cerebrovascular diseases that may occur later in life. Other studies suggested that changes in subclinical carotid atherosclerosis over 5 years were associated with a longitudinal decrease in cerebral glucose metabolism in parietotemporal brain regions, suggesting that the effect of carotid atherosclerosis on cerebral glucose consumption is associated with that of CVRFs [93].

Recently, an updated SCORE model, known as SCORE2, was established to increase the 10-year prediction of CVD risk in individuals across Europe [39]. SCORE2 is more accurate in estimating the impact of CVD and other risk factors on the incidence of fatal and nonfatal CVD events in various categories of patients, for example, subjects with DM-2, older age, etc. [36]. SCORE2-OP is the most recent cardiovascular risk score and is employed to estimate the risk of CVD in people over 70 years in four geographic risk regions [39].

A study on 429,033 participants without dementia, which used the SCORE2 risk algorithm, determined that subjects with higher SCORE2 values had an increased risk of dementia, Alzheimer’s disease, vascular disease, and all-cause mortality. The study concluded that the results of SCORE2 may be useful in predicting the risk of dementia, Alzheimer’s disease, vascular disease, and all-cause mortality in the European population. The results of this study also demonstrated that individuals with a higher SCORE2 had an increased risk of developing dementia [94]. In our study, we observed that SCORE2 values as well as parameters characterizing CD and dementia, depression, and quality of life were more altered in patients with DM-2 and/or AF compared to those without any of these two pathologies.

One study evaluated 19,114 participants aged over 65 years without known CVD or dementia. Over a follow-up period of 6.4 years, 850 people developed dementia and 4352 CD. Men and women in the highest SCORE2-OP tertile had a 64% and 60% increased risk of dementia compared to the lowest tertile [95]. In our study, we documented mildly positive but statistically significant correlations between SCORE2 values and GDS-15 (*p* = 0.011) and negative ones with MMSE, MoCA, ADL, and IADL (*p* = 0.002, *p* = 0.004, *p* = 0.048, and *p* = 0.011). We documented weakly positive but significant correlations between SCORE 2 values and NT pro-BNP levels (*p* = 0.029). In our study, when considering patients with DM-2 and/or AF and HCVRF patients, the odds of developing MMSE < 27 or MMSE < 24 increased in a statistically significantly parallel manner with SCORE2. If new elements of CVRF appear (SCORE2 increases by 1%), the risk of CD impairment increases (with 2.4% in AF and/or DM-2 patients) and also augments (with 3.1% in all CVRF patients) the odds of MMSE between 27 and 24. An increase of 1% in SCORE2 was associated with an increase of 4.3% in the odds of MMSE < 24 (dementia) in patients with DM-2 and/or AF, while an increase of 1% in SCORE2 was associated with an elevation of 4.7% in the odds of MMSE < 24 (dementia) in all CVRF patients. Therefore, adequate control of CVRF, especially in patients with associated DM-2 and AF, can reduce the risk of CD, and in these populations, lifestyle changes and statin treatment should be initiated earlier. Still, this effort must be continued to obtain an even better control of the lipid profile during follow-up, which could help delay the onset of CD.

Thus, taking into account that patients suffering from atherosclerotic CVD and/or neurologic pathologies also have an unfavorable VHCVR profile, DM-2, and AF and are more prone to developing cognitive impairment, the additional development of CD and/or dementia will further worsen their health status, self-care capacity, and the quality of life. Therefore, an integrated approach is recommended by addressing them as soon as possible through a multidisciplinary team, including specialists in neurology, psychiatry, psychology, and cardiology, for their comprehensive evaluation, with the help of neuropsychological tests, for a potential onset of CD in the attempt to slow down progression towards dementia. Also, considering that in patients with DM-2 and AF, any augmentation of depressive elements may accentuate CD and dementia and alter their quality of life, it is important to treat the depressive symptoms accurately to slow down the evolution of CD.

One of the limitations of our study might be related to the smaller sample size of patients included in the study, thus limiting the statistical power to detect group effects. Another limitation results from the fact that it is a single-center study, and our patients were not studied longitudinally over time to follow-up the evolution of these parameters assessed by neuropsychological scales. Also, we did not analyze the relationship between CD and silent or short episodes of AF or the effects of various therapies, for example, anticoagulant treatment in patients with AF. Larger cohort studies in which cognitive tests are employed to evaluate patients with AF and DM-2 are needed. It is also necessary to investigate interventions such as lifestyle changes and drugs to prevent or delay CD in these patients.

## 5. Conclusions

AF, DM-2, and CD are major health problems expected to increase exponentially in the general population. A worse VHCVR profile, as assessed through SCORE2, especially in patients with DM-2 and/or AF, can increase the risk of CD. Our findings indicate comorbidities such as DM-2 and AF as ideal targets for preventive measures to slow down and delay the development of CD and its progression to dementia. We determined that even slight elevations of SBP and DBP can alter cognitive function. Another conclusion of our study was that if depressive symptoms and the quality of life improve, the risk of CD decreases by 44.0% and that of dementia by 36.6%. Patients with DM-2 and/or AF for whom SCORE2 values indicate VHCVR should be screened for CD with the help of neuropsychological tests in an attempt to diagnose CD earlier and to slow down its progression to dementia. Any measurements to treat depression and to increase the quality of life must be considered in these patients. Our results suggest that patients with DM-2 and/or AF should be followed-up and evaluated regularly by a neurologist to detect the onset of CD and dementia. Further research is needed to validate these results in different populations with various associations of risk factors and comorbidities.

## Figures and Tables

**Table 1:** Associated cardiovascular diseases and risk factors in all four groups (counts and associated percentages).

Patients’ Demographic Data	Group A 63 P with DM-2	Group B 60 P with DM-2 and AF	Group C 61 P with AF	Group D 64 Controls	*p*-Value	*p*1 Between A and B	*p*2 Between A and C	*p*3 Between B and C	*p*4 Between A and D	*p*5 Between B and D	*p*6 Between C and D
Sex: male	36 (57.10%)	29 (48.30%)	20(32.80%)	28(43.80%)	0.053	0.328	0.006	0.082	0.131	0.609	0.208
female	27 (42.90%)	31 (51.70%)	41(67.20%)	36 (56.20%)	0.053	0.328	0.006	0.082	0.131	0.609	0.208
Systemic hypertension (SH)	57/90.50	56/93.30	56/91.80	55/85.90	0.535	0.562	0.795	0.748	0.428	0.179	0.299
Chronic coronary syndrome (CCS)	18/28.60	20/33.30	19/31.10	12/18.80	0.274	0.571	0.756	0.799	0.196	0.064	0.110
Peripheral arterial disease (PAD)	5/10.00	3/5.00	7/11.50	3/4.70	0.434	0.509	0.505	0.196	0.451	0.935	0.162
Minor stroke	6/8.00	8/13.30	7/11.50	5/7.80	0.769	0.506	0.723	0.757	0.732	0.316	0.487
Lacunar stroke	9/14.00	10/16.70	7/11.50	6/9.40	0.641	0.715	0.641	0.411	0.391	0.226	0.701
Chronic kidney disease:	38/60.30	43/71.70	38/62.30	33/51.60	0.149	0.185	0.821	0.273	0.320	0.022	0.226
CKD stage 1	8/12.70	9/15.00	9/14.80	11/17.20	0.918	0.712	0.739	0.970	0.620	0.741	0.711
CKD stage 2	11/17.50	13/21.70	11/18.00	10/15.60	0.851	0.556	0.934	0.616	0.781	0.387	0.719
CKD stage 3	16/25.40	16/26.70	15/24.60	10/15.60	0.438	0.873	0.917	0.794	0.172	0.131	0.210
CKD stage 4	3/4.80	5/8.30	3/4.90	2/3.10	0.621	0.422	0.968	0.450	0.635	0.209	0.609
Chronic heart failure	52/82.50	54/90.00	51/83.60	50/78.10	0.359	0.231	0.874	0.299	0.532	0.072	0.437
(CHF) NYHA 1	5/7.90	4/6.70	4/6.60	7/10.90	0.787	0.787	0.767	0.981	0.563	0.403	0.388
NYHA 2	31/49.20	31/51.70	30/49.20	30/46.90	0.963	0.785	0.998	0.784	0.793	0.594	0.797
NYHA 3	13/20.60	15/25.00	14/23.00	11/17.20	0.742	0.564	0.755	0.792	0.620	0.286	0.421
NYHA 4	3/4.80	4/6.70	3/4.90	2/3.10	0.839	0.649	0.968	0.680	0.635	0.358	0.609
Obesity: BMI > 30 Kg/m^2^	26/41.30	26/43.3	19/31.10	11/17.20	0.007	0.817	0.241	0.166	0.003	0.001	0.068
Obesity gr 1,	11/17.50	10/16.70	10/16.40	2/3.10	0.051	0.907	0.874	0.968	0.008	0.011	0.012
Obesity gr 2,	7/11.10	6/10.00	2/3.30	3/4.70	0.248	0.841	0.093	0.137	0.179	0.255	0.688
Obesity gr 3	8/12.70	10/16.70	7/11.50	6/9.40	0.663	0.534	0.835	0.411	0.550	0.226	0.701
Smoking	23/36.50	16/26.70	11/18.00	26/40.60	0.029	0.241	0.021	0.254	0.634	0.101	0.006
Hyperlipemia	28/44.40	37/61.10	38/62.30	35/54.70	0.158	0.056	0.046	0.943	0.248	0.431	0.388

Legend: AF—atrial fibrillation; BMI—body mass index; CCS—chronic coronary syndrome; CKD—chronic kidney disease; SH—systemic hypertension; CHF -chronic heart failure; NYHA—New York Heart Association; P—patient, PAD—peripheral artery disease; DM-2—diabetes mellitus type 2; *p*—statistical significance according to the chi-square test.

**Table 2:** Clinical, hemodynamic, and laboratory data in all four groups.

Patients’ Demographic Data	Group A 63 P with DM-2	Group B 60 P with DM-2 and AF	Group C 61 P with AF	Group D 64 P Controls	*p*1 Between A and B	*p*2 Between A and C	*p*3 Between B and C	*p*4 Between A and D	*p*5 Between B and D	*p*6 Between C and D
Mean age	67.97 ± 10.39	73.03 ± 9.05	72.21 ± 8.24	68.67 ± 10.01	0.005	0.013	0.603	0.694	0.012	0.033
WC (cm)	94.65± 10.49	96.50 ± 9.20	92.67± 7.35	90.66 ± 11.18	0.302	0.228	**0.013**	0.040	0.002	0.239
BMI (kg/m^2^)	32.44 ± 6.87	31.82 ± 7.31	30.35 ± 6.44	28.21 ± 7.21	0.628	0.084	0.244	0.001	0.007	0.083
HR (b/min)	76.48 ± 13.74	77.78 ± 18.16	75.08 ± 16.88	71.84 ± 12.39	0.653	0.616	0.399	0.048	0.035	0.222
SBP (mmHg)	133.10 ± 23.42	133.92 ± 22.51	130.41 ± 21.76	127.42 ± 15.58	0.843	0.509	0.386	0.110	0.063	0.381
DBP (mmHg)	79.52 ± 12.91	80.50 ± 14.94	78.93 ± 14.89	74.22 ± 15.22	0.700	0.814	0.565	0.036	0.022	0.083
SCORE2 (%)	39.10 ± 12.78	43.12 ± 12.46	38.54 ± 14.25	31.17 ± 14.58	0.080	0.816	0.062	0.001	<0.001	0.005
Laboratory data										
Total chol. (mg/dL)	188.58 ± 55.43	174.38 ± 58.92	173.37 ± 58.2	192.67 ± 64.42	0.171	0.139	0.925	0.702	0.102	0.081
LDL chol (mg/dL)	130.66 ± 50.50	127.36 ± 53.48	135.81 ± 60.9	137.48 ± 52.66	0.726	0.610	0.419	0.458	0.291	0.871
HDL chol. (mg/dL)	53.65 ± 24.72	44.03 ± 11.36	44.40 ± 15.50	49.03 ± 15.20	0.007	0.014	0.879	0.208	0.040	0.095
Triglycerides (mg/dL)	126.52 ± 58.13	127.93 ± 50.80	128.70 ± 51.98	134.42 ± 62.64	0.887	0.826	0.934	0.463	0.526	0.579
Basal plasma glucose (mg/dL)	110.79 ± 23.18	114.87 ± 40.99	107.1 ± 20.41	105.52 ± 16.00	0.496	0.348	0.192	0.137	0.103	0.631
TyG index	4.71 ± 0.27	4.74 ± 0.23	4.76 ± 0.23	4.68 ± 0.28	0.552	0.289	0.620	0.525	0.210	0.090
Uric acid (mg/dL)	5.89 ± 1.63	6.43 ± 1.30	6.21 ± 1.34	6.37 ± 1.76	0.047	0.229	0.384	0.110	0.853	0.571
eGRF (ml/min)	53.98 ± 13.43	53.86 ± 14.71	59.54 ± 17.94	63.23 ± 19.26	0.960	0.053	0.059	0.002	0.003	0.269
NT-pro-BNP (pg/mL)	2844.5 ± 844.6	3008.5 ± 848.4	2967.7 ± 638.4	2736.4 ± 845.5	0.285	0.360	0.766	0.472	0.076	0.086

P—patient; WC—waist circumference; BMI—body mass index; SBP—systolic blood pressure; DBP—diastolic blood pressure; HR—Legend: HR—heart rate; DM-2—diabetes mellitus type 2; AF—atrial fibrillation; SCORE2—prediction algorithm to estimate 10-year fatal and non-fatal CVD risk; LDL chol.—low-density lipoprotein; HDL chol.—high-density lipoprotein; eGRF—estimated glomerular filtration rate; TyG—triglyceride glucose index; NT-pro-BNP—N-terminal pro–B-type natriuretic peptide; *p*—statistical significance according to the unpaired *t*-test.

**Table 3:** Results of IMT and ABI measurements in all 4 groups of patients.

Subclinical Atherosclerosis Parameters	Group A 63 P with DM-2	Group B 60 P with DM-2 and AF	Group C 61 P with AF	Group D 64 Controls	*p*1 Between A and B	*p*2 Between A and C	*p*3 Between B and C	*p*4 Between A and D	*p*5 Between B and D	*p*6 Between C and D
IMT (mm) left	0.67 ± 0.29	0.77 ± 0.26	0.72 ± 0.25	0.65 ± 0.26	0.068	0.361	0.308	0.644	0.016	0.143
IMT (mm) right	0.67 ± 0.32	0.73 ± 0.29	0.69 ± 0.25	0.65 ± 0.29	0.263	0.697	0.390	0.678	0.105	0.374
ABI left	1.09 ± 0.12	1.06 ± 0.10	1.08 ± 0.12	1.10 ± 0.09	0.173	0.678	0.353	0.622	0.037	0.337
ABI right	1.06 ± 0.09	1.09 ± 0.19	1.08 ± 0.17	1.11 ± 0.11	0.280	0.423	0.795	0.006	0.457	0.263

Legend: IMT—intima–media thickness, ABI—Ankle Brachial Index; *p*—statistical significance according to the unpaired *t*-test.

**Table 4:** TTE parameters of left ventricle systolic and diastolic function in patient groups.

Echocardiographic Parameters	Group A 63 P with DM-2	Group B 60 P with T2DM and AF	Group C 61 P with AF	Group D 64 Controls	*p*1 Between A and B	*p*2 Between A and C	*p*3 Between B and C	*p*4 Between A and D	*p*5 Between B and D	*p*6 Between C and D
IVS (mm)	11.98 ± 1.90	11.97 ± 1.87	12.14 ± 2.18	11.89± 2.50	0.983	0.661	0.648	0.810	0.825	0.542
LA (mm)	40.69 ± 5.95	42.98 ± 6.89	41.3 ± 7.64	39.71 ± 5.18	0.051	0.620	0.209	0.325	0.003	0.174
LVPW (mm)	12.18 ± 2.57	11.58 ± 2.03	12.23 ± 2.62	12.37 ± 2.76	0.157	0.917	0.135	0.683	0.075	0.763
LVESD (mm)	22.55 ± 5.94	24.60 ± 7.28	24.18 ± 6.51	24.82 ± 6.68	0.092	0.150	0.739	0.045	0.856	0.584
LVEDD (mm)	45.06 ± 5.10	46.88 ± 6.49	46.78 ± 6.25	45.09 ± 3.82	0.088	0.096	0.934	0.970	0.062	0.069
LVESV (mL)	33.79 ± 14.9	38.28 ± 13.79	40.14 ± 13.79	33.75 ± 11.86	0.086	0.015	0.459	0.985	0.053	0.006
LVEDV (mL)	72.23 ± 17.3	77.35 ± 17.04	76.01 ± 19.64	72.40 ± 15.63	0.101	0.258	0.691	0.954	0.096	0.259
LVEF (%)	57.17 ± 6.38	55.61 ± 7.33	56.52 ± 8.51	58.35 ± 5.55	0.213	0.632	0.531	0.267	0.020	0.154
E (m/s)	0.73 ± 0.15	0.72 ± 0.20	0.71 ± 0.16	0.69 ± 0.18	0.923	0.449	0.578	0.195	0.298	0.571
TRVmax (m/s)	2.26 ± 0.42	2.50 ± 0.57	2.45 ± 0.54	2.28 ± 0.44	0.011	0.034	0.643	0.860	0.018	0.052
sPAP (mmHg)	35.46 ± 10.8	40.96 ± 12.59	40.85 ± 11.84	36.45 ± 11.62	0.011	0.009	0.959	0.619	0.041	0.038

Legend: LA—left atrial; IVS: interventricular septum; LVPW—left ventricular posterior wall; LVEDD—left ventricular end-diastolic diameter; LVESD—left ventricular end-systolic diameter; LVEDV—left ventricular end-diastolic volume; LVESV—left ventricular end-systolic volume; E—peak early diastolic transmitral flow velocity; LVEF—left ventricular ejection fraction; TRVmax—maximal tricuspid velocity; sPAP—pulmonary systolic arterial pressure; *p*—statistical significance according to the unpaired *t*-test.

**Table 5:** Results of the neuropsychological tests in all 4 groups of patients.

Neuropsychological Tests	Group A 63 P with DM-2	Group B 60 P with DM-2 and AF	Group C 61 P with AF	Group D 64 Controls	*p*1 Between A and B	*p*2 Between A and C	*p*3 Between B and C	*p*4 Between A and D	*p*5 Between B and D	*p*6 Between C and D
MMSE	26.46 ± 4.39	25.00 ± 4.80	25.85 ± 3.91	27.81 ± 3.48	0.081	0.418	0.287	0.058	<0.001	0.004
MoCA	23.52 ± 5.16	22.83 ± 5.22	23.75 ± 5.01	25.30 ± 4.43	0.463	0.802	0.325	0.040	0.005	0.070
ADL	9.43 ± 0.97	9.12 ± 1.23	9.26 ± 1.15	9.72 ± 0.82	0.123	0.389	0.504	0.073	0.002	0.012
IADL	6.68 ± 1.81	6.52 ± 1.77	6.85 ± 1.48	7.30 ± 1.31	0.609	0.568	0.260	0.031	0.006	0.079
GDS-15	6.69 ± 2.24	7.38 ± 2.46	7.11 ± 2.01	6.00 ± 2.20	0.110	0.280	0.513	0.080	0.001	0.004

Legend: P—patients, MMSE—Mini-Mental State Examination Scale; MoCA—Montreal Cognitive Assessment Scale; ADL—Activities of Daily Living Score; IADL—Instrumental Activities of Daily Living Score; GDS-15—Geriatric Depression Scale 15 questions; *p*—statistical significance according to the unpaired *t*-test.

**Table 6:** Correlation between SCORE2, LVEF, age, SBP, DBP, and NT pro-BNP levels and MMSE, MoCA, ADL, IADL, and GDS-15.

Parameter	MMSE	MoCA	ADL	IADL	GDS-15
SCORE2					
r	−0.200	−0.180	−0.161	−0.126	0.160
95% CI	−0.318; −0.076	−0.299; −0.056	−0.281; −0.036	−0.247; −0.001	0.035; 0.280
*p*	0.002	0.004	0.011	0.048	0.011
**LVEF**					
r	0.119	0.122	0.343	0.302	−0.309
95% CI	−0.006; 0.240	−0.003; 0.243	0.225; 0.451	0.182; 0.413	−0.420; −0.189
*p*	0.062	0.054	<0.001	<0.001	<0.001
**Age**					
r	−0.387	−0.178	−0.282	−0.259	0.277
95% CI	−0.492; −0.271	−0.297; −0.054	−0.395; −0.161	−0.373; −0.137	0.156; 0.390
*p*	<0.001	0.005	<0.001	<0.001	<0.001
**SBP**					
r	−0.468	−0.473	−0.174	−0.319	0.405
95% CI	−0.564; −0.359	−0.569; −0.364	−0.293; −0.050	−0.429; −0.199	0.291; 0.508
*p*	<0.001	<0.001	0.006	<0.001	<0.001
**DBP**					
r	−0.474	−0.470	−0.280	−0.243	0.390
95% CI	−0.570; −0.365	−0.566; −0.361	−0.393; −0.159	−0.358; −0.120	0.275; 0.494
*p*	<0.001	<0.001	<0.001	<0.001	<0.001
**NT pro-BNP**					
r	−0.179	−0.125	−0.176	−0.128	0.058
95% CI	−0.298; −0.055	−0.246; −0.001	−0.295; −0.054	−0.249; −0.003	−0.067;−0.181
*p*	0.005	0.049	0.005	0.044	0.365

Legend: MMSE—Mini-Mental State Examination Scale; MoCA—Montreal Cognitive Assessment Scale; ADL—Activities of Daily Living Score, IADL—Instrumental Activities of Daily Living Score; GDS-15—Geriatric Depression Scale 15 questions; SCORE2—prediction algorithm to estimate 10-year fatal and non-fatal CVD risk, LVEF—left ventricular ejection fraction, SBP—systolic blood pressure; DBP—diastolic blood pressure; NT pro-BNP—N-terminal pro–B-type natriuretic peptide; r—Spearman’s correlation coefficient, 95% CI = 95% confidence interval estimated using Bonnett and Wright’s method.

**Table 7:** Prognostic factors for developing CD, quantified by MMSE between 24 and 27 for SCORE2 in all 248 CVRFs patients and 184 patients with DM-2 and/or AF (group A with DM-2, group B with DM-2 and AF, and group C with AF).

Patients with MMSE < 27 n (%) *		OR (95% CI)	*p*-Value
248 HCVRFs patients			
SCORE2	84 (33.87%)	1.031 (1.011; 1.052)	0.003
184 patients with DM-2 and/or AF			
SCORE2	75 (40.76%)	1.024 (1.001;1.048)	0.044

Legend: OR—odds ratio, 95% CI—95% confidence interval. * Percentages based on the total number of patients with DM-2 and or AF (184 patients) or the total number of patients with CVRFs included in the study.

**Table 8:** Prognostic factors for presenting CD, quantified by MMSE between 24 and 27, in 248 VHCVR patients or in 184 patients with DM-2 and/or AF (group A, B, and C).

Patients with MMSE < 27 n (%) *		OR (95% CI)	*p*-Value
248 CVRFs patients			
Age (years)	84 (33.87%)	1.123 (1.076; 1.173)	<0.001
SBP (mmHg)	84 (33.87%)	1.025 (1.005; 1.046)	0.016
DBP (mmH)	84 (33.87%)	1.052 (1.022; 1.084)	0.001
184 patients with DM-2 and/or AF			
Age (years)	75 (40.76%)	1.140 (1.081; 1.201)	<0.001
SBP (mmHg)	75 (40.76%)	1.023 (1.001; 1.046)	0.042
DBP (mmHg)	75 (40.76%)	1.080 (1.039; 1.123)	<0.001

Legend: OR—odds ratio, 95% CI—95% confidence interval. * Percentages based on the total number of patients with DM-2 and or AF (184 patients) or the total number of patients with CVRFs included in the study.

**Table 9:** Prognostic factors for presenting CD, quantified by MMSE between 27 and 24, for ADL and GDS-15 in all 248 CVRFs patients (group A with DM-2, group B with DM-2 and AF, group C with AF, and group D as controls) or in 184 patients with DM-2 and/or AF (group A with DM-2, group B with DM-2 and AF, and group C with AF).

	Patients with MMSE < 27 n (%) *	OR (95% CI)	*p*-Value
248 CVRFs patients			
ADL	84 (33.87%)	0.485 (0.352; 0.667)	<0.001
GDS-15	84 (33.87%)	1.374 (1.176; 1.606)	<0.001
184 patients with DM-2 and/or AF			
ADL	75 (40.76%)	0.560 (0.396; 0.790)	0.001
GDS-15	75 (40.76%)	1.363 (1. 142; 1.627)	0.001

Legend: OR—odds ratio, 95% CI—95% confidence interval. * Percentages based on the total number of patients with DM-2 and/or AF (184 patients) or the total number of patients with CVRFs included in the study.

**Table 10:** Prognostic factors for presenting dementia, quantified by MMSE < 24 for SCORE2, in all 248 VHCVR patients or 184 patients with DM-2 and/or AF (group A, B, and C).

Patients with MMSE < 24 (Dementia) n (%) *		OR (95% CI)	*p*-Value
**248 CVRFs patients**			
SCORE2 (%)	55 (22.18%)	1.047 (1.021; 1.074)	<0.001
**184 patients with DM-2 and/or AF**			
SCORE2 (%)	50 (27.17%)	1.043 (1.013;1.074)	0.004

Legend: OR—odds ratio, 95% CI—95% confidence interval. * Percentages based on 184 patients with DM-2 and/or AF or the total number of study patients with HCVRFs.

**Table 11:** Multiple logistic regression analysis for risk of MMSE < 24. Prognostic factors for dementia, quantified by MMSE < 24 for age and DBP in all 248 VHCVR patients (group A, B, C, and D) or 184 patients with DM-2 and/or AF (group A, B, and C).

Patients with MMSE < 24 (Dementia) n (%) *		OR (95% CI)	*p*-Value
248 CVRFs patients			
Age (years)	55 (22.18%)	1.154 (1.096; 1.214)	<0.001
DBP (mmHg)	55 (22.18%)	1.077 (1.048; 1.106)	<0.001
184 patients with DM-2 and/or AF			
Age (years)	50 (27.17%)	1.147 (1.083; 1.214)	<0.001
DPB (mmHg)	50 (27.17%)	1.095 (1.058; 1.133)	<0.001

Legend: OR—odds ratio, 95% CI—95% confidence interval. * Percentages based on the total number of patients with DM-2 and or AF (184 patients) or the total number of patients with CVRFs included in the study.

**Table 12:** Multiple logistic regression analysis for risk of MMSE < 24. Prognostic factors for presenting dementia, quantified by MMSE < 24, for ADL and GDS-15 in all 248 CVRFs patients (group A, B, C, and D) or 184 patients with DM-2 and/or AF (group A, B, and C).

Patients with MMSE < 24 (Dementia) n (%) *		OR (95% CI)	*p*-Value
248 CVRFs patients			
ADL	55 (22.18%)	0.508 (0.372; 0.694)	<0.001
GDS-15	55 (22.18%)	1.521 (1.256; 1.841)	<0.001
184 patients with DM-2 and/or AF			
ADL	50 (27.17%)	0.634 (0.454; 0.884)	0.007
GDS-15	50 (27.17%)	1.574 (1.263; 1.961)	<0.001

Legend: OR—odds ratio, 95% CI—95% confidence interval. * Percentages based on the total number of patients with DM-2 and/or AF (184 patients) or the total number of patients with CVRFs included in the study.

## Data Availability

The data supporting the reported results can be obtained from the first author of this manuscript, Marius Militaru, upon request.
